# The expression and function of KCNQ potassium channels in human chorionic plate arteries from women with normal pregnancies and pre-eclampsia

**DOI:** 10.1371/journal.pone.0192122

**Published:** 2018-03-26

**Authors:** Xiaohong Wei, Yujiao Zhang, Benlan Yin, Jing Wen, Jun Cheng, Xiaodong Fu

**Affiliations:** 1 Department of Gynecology and Obstetrics, the Affiliated Hospital of Southwest Medical University, Luzhou, Sichuan, China; 2 Key Laboratory of Medical Electrophysiology of Ministry of Education, Collaborative Innovation Center for Prevention and Treatment of Cardiovascular Research, Southwest Medical University, Luzhou, Sichuan, China; Tel Aviv University Sackler Faculty of Medicine, ISRAEL

## Abstract

Pre-eclampsia is associated with altered maternal and placental vascular reactivity. Kv7 channels (encoded by KCNQ 1–5 genes) are a potential contributor to the regulation of vascular tone in CPAs (chorionic plate arteries) during normal pregnancy. The aim of this study is to establish the expression profile of KCNQ subunits in CPAs taken from women with preeclampsia or normotensive women and to examine the functional relevance of the Kv7 channels on an altered expression profile of KCNQ subunits. The effects of Kv7 channel modulators on CPAs were investigated by tension measurement. Quantitative PCR experiments were used to analyze the expression of KCNQ genes. Western blotting and immunofluorescence were both used to analyze the protein expression of Kv7 channels. Finally, in CPAs from normotensive women, the Kv7 channel blocker XE991 increased arterial basal tone and U46619-induced contraction, and pre-contracted CPAs (10^−7^ M U46619) exhibited significant relaxation following treatment with Retigabine(Kv7.2–7.5 activator) and BMS-204352(Kv7.2–7.5 activator). However, ICA-27243(selective KCNQ2 and KCNQ3 activator) and ML277(selective KV7.1 activator) had no significant effect on tension in the pre-contracted CPAs. Conversely, compared with CPAs from normotensive women, the effects of XE991 on basal tone and agonist (U46619)-induced contractions in CPAs from women with preeclampsia were markedly attenuated. Moreover, the relaxation effects of Retigabine and BMS-204352 on pre-contracted CPA vessels from women with pre-eclampsia were also markedly down-regulated. Interestingly, the relaxation ability of ICA-27243 in pre-contracted CPA vessels in women with pre-eclampsia was enhanced. The mRNA of KCNQ3 was specifically up-regulated, whereas those for KCNQ4 and KCNQ5 were down-regulated in CPAs from women with pre-eclampsia compared with those in normotensive women. Similar observations were found in a subsequent analysis of protein expression of KCNQ genes 3–5. Thus, down-regulated Kv7 channel function in tension regulation of CPAs in women with pre-eclampsia could be associated with considerably altered expression profiles of Kv7 subunits.

## Introduction

Pre-eclampsia is currently the most common and significant complication in obstetrics, complicating 3–5% of all pregnancies [1]. Preeclampsia, hemorrhage and infection are the three leading causes contributing to maternal and perinatal morbidity and mortality worldwide [1–2]. The essential clinical presentation of preeclampsia traditionally entails new-onset hypertension and proteinuria occurring after 20 weeks of gestation in a previously normotensive woman. However, maternal multiorgan dysfunction is also included in the manifestations of this disorder, according to the new definition [2–3]. This disorder is universally combined with fetal growth restriction and preterm delivery. Women with complicated preeclampsia and their neonates are at increased risk of hypertension, metabolic disorders, cardiovascular disease and cardiovascular death in their future lives [4–5].

The pathophysiology of preeclampsia is complex. Placental dysfunction and impaired remodeling of the spiral arteries are almost certainly contributors to this disease [6–8]. Reduced uteroplacental arterial flow and irregular placental perfusion result in hypoxia or reoxygenation episodes [9], which lead to placental oxidative stress and dysfunction [10] and subsequently a generalized hyper inflammatory state compared with normal pregnancy [11]. The excessive inflammatory response results in exaggerated endothelial activation and altered maternal and placental vascular reactivity [12].

The fetal-placental circulation mediates exchange of nutrients and waste products between the maternal and perinatal systems and thereby supplies adequate oxygen/nutrients for normal fetal development [13]. Chorionic plate arteries (CPAs) return deoxygenated blood from the fetus to the feto-maternal interface where gas/nutrient exchange occurs, playing a vital role in the fetal-placental circulation [13, 14]. The exaggerated endothelial activation and altered placental vascular reactivity of CPAs in women with pre-eclampsia increase vascular tone and reduce fetoplacental blood flow preceding onset of clinical manifestations such as fetal growth restriction and preterm [15].

It’s accepted that Potassium channels are the predominant mediators of modulation of smooth muscle contractility due to their decisive role in changes in cell membrane potential (Vm) [16,17]. The depolarization of vascular smooth muscle cells (VSMCs), resulting from inhibition of K+ channels, leads to a calcium (Ca^2+^) influx via L-type voltage-dependent Ca^2+^ channels and subsequent vasocontraction [18]. Several reviews over the past decades have implicated K+ channels in regulating excitation-contraction coupling of CPAs, including K_v_, K_ATP_, K_2_P and K_Ca_ channels [19]. Kv7 channels (encoded by KCNQ genes), subfamily of voltage-gated potassium channel complexes, have recently become a hot topic because of their important contributions to the regulation of contractility of VSMCs. The first report that Kv7 channels in VSMCs was in the mouse portal vein[20].Yeung and Greenwood implicated their important contribution to resting membrane voltage and smooth muscle excitability [21]. Kv7 channels were also shown to be expressed in numerous blood vessels, including pulmonary arteries, mesenteric arteries and coronary arteries of rats and mice, as well as in rat middle cerebral and basilar arteries, where they play an important role in the regulation of vascular reactivity [22–24]. The broad spectrum Kv7 blockers, linopirdine and XE991, cause vascular contraction of a number of blood vessels including the mesenteric, pulmonary and cerebral arteries, whereas the activator retigabine relaxes pre-constricted artery vessels [23–25]. Further observations have shown the expression and function of variable Kv7 channel isoforms in human visceral fat and mesenteric arteries [26]. More recently, CPAs were found to express Kv7 channels that constricted in response to linopirdine and were relaxed by the Kv7.2–5 specific activators flupirtine, Retigabine and S-1 in normotensive patients [27]. Considering their excitability-regulating abilities, Kv7 channels could be very promising new therapeutic targets for treatment of pre-eclampsia cases where compromised fetoplacental blood flow is apparent, but their pathophysiological role in these disorders has not yet been reported.

Hence, the aim of this study was to extend previous work on Kv7 channels in CPAs by establishing the expression profiles of KCNQ subunits in CPAs taken from women with pre-eclampsia, as well as normotensive controls, and examining the functional relevance of the KCNQ channels on altered expression profiles.

## Materials and methods

This study was approved by the ethics committee of Luzhou Medical College, and written informed consents were obtained from all women prior to initiation of delivery. A total of 46 cases were recruited from May 2015 to May 2016 by either vaginal delivery or caesarean section, including 26 cases diagnosed with preeclampsia as the experimental group (pre-eclampsia, PE) and 20 cases with normotensive pregnancy serving as a control group (normotensive pregnancy, NP). The clinical characteristics of all participating cases were obtained, including maternal age, primipara, blood pressure, proteinuria, birth weight, gestational age at delivery (wk), number of deliveries < 37 wk (n [%]) and proportion of FGR [28] (Table 1). Cases of pre-eclampsia were admitted according to the American College of Obstetricians and Gynecologists’ (ACOG) guidelines [29–30]. All cases with complications of pregnancy (including cardiovascular disease, severe liver and kidney disease, immune system disease, endocrine disease, cancer, mental or cognitive disorders), obstetricalcomplications (gestational diabetes, multiple pregnancies, placental abruption and placenta previa) and other relevant medical histories (smoking, drinking, a history of drug use during pregnancy history, etc.) were excluded. Small tissue samples, approximately 2×2×2 cm^3^, were immediately taken from newly delivered placentas at a location midway between the root of the umbilical cord and the border of the placenta and placed into ice-cold normal Tyrode’s solution (in mM: 127 NaCl, 5.9 KCl, 1.2 MgCl_2_, 2.4 CaCl_2_, 12 Glucose, 10 HEPES; pH 7.4 with NaOH). Then, appropriately sized CPA branches were identified and surrounding connective tissues were removed under a stereoscopic microscope in ice-cold normal Tyrode’s solution.

**Table 1:** Clinical characteristics of all participating subjects.

Parameter	Normal pregnancy(N = 20)	Preeclampsia(N = 26)
Matemal age	28.3±3.9	30.5±7.1*
Primipara,n(%)	12(60)	10(38.5)
Maximum systolic(mm Hg)	114.5±9.8	170.8±15.3*
Maximum diastolic(mm Hg)	75.9±9.0	110.9±143*
Proteinuria, g/L(Maximum, minimum)	0.04(0.01, 0.23)	3.1 (0.14, 11.0)
Cesarean section, n (%)	3 (15)	11(42.3)
Gestational age at delivery <37 wk, n (%)	0	15(57.7)
Birth weight	3.4 (2.61 to 4.18)	2.33 (1.1 to 5.12)

Data are presented as the mean±SD or median (interquartile range), except for proteinuria (median [minimum, maximum]), Primipara [n(%)], caesarean sections [n(%)], and gestational age at delivery <37 wk [n(%)].

*P<0.01 between normal and pre-eclamptic pregnancies.

### Measurement of vascular tension in vitro

The CPAs (lumen diameter ~300 μm) were cut into rings approximately 1.5–2 mm in length and mounted equidistantly in an organ chamber filled with 5 ml oxygenated (aerated with 95% O_2_, at 37°C) normal Tyrode’s solution. As described previously, the initial resting tension was adjusted according to DMT (120CW Confocal Wire Myograph, Denmark) normalization for 60 min[31]. Post-equilibration, contractile viability was assessed with high K^+^ (80 mM) Tyrode’s solution (equimolar substitution of NaCl with KCl to achieve the K^+^ concentration of 80 mM). When these procedures were finished, the artery rings were washed and recuperated for at least 30 min before the next measurement [31].

The effect of XE991 (10^−7^–10^−4^ M) was assessed on basal arterial tone and in the presence of a contractile agonist (U46619_,_10^−10^–3×10^−6^ M) in all arteries from normotensive or pre-eclamptic women. Then artery segments from normotensive or pre-eclamptic women were treated with 10–7M U46619 to attain a stable tension, after that, cumulative dose-dependent relaxation was assessed by applying a Kv7 channel activating drug(or DMSO as vehicle control) at increasing concentrations ranging from 10–9 to 10–4M.Structurally disparate Kv7 channel activators are ML277, retigabine, BMS-204352, and ICA-27243.

ML277, Retigabine, (S) BMS-204352, XE991 and U46619 were obtained from Sigma-Aldrich (Gillingham, UK), and ICA-27243 was purchased from Alomone Labs (Jerusalem Bio Park (JBP), Israel)(Table 2).

**Table 2:** Information of relative drugs.

Common Name	Chemical Name	Reported Action
XE991	10,10-bis(4-pyridinylmethyl)- 9(10H)-anthracenone)	Kv7 channel broad spectrum blocker^[26]^
U46619	9,11-Dideoxy-11α,9α- epoxymethanoprostaglandin F_2α_	contractile agonist
ML277	4-Methoxyphenyl)-1,3-thiazol-2-yl]-1-(4-methylphenyl)sulfonylpiperidine-2-carboxamide(R)-N-(4-(4-Methoxyphenyl) thiazol-2-yl)-1-tosylpiperidine-2-carboxamide	selective KV7.1 activator^[32]^
Retigabine	Ezogabine N-(2-Amino-4-(4-fluorobenzylamino)phenyl) carbamic acid ethyl ester	Kv7.2–7.5 activator^[27]^
BMS-204352	(3S)-(+)-(5-Chloro-2-methoxyphenyl)-1,3-dihydro-3–fluoro-6-(trifluoromethyl) -2H- indole-2-one	Kv7.2–7.5 activator^[27]^
ICA-27243	N-(6-chloro-pyridin-3-yl)-3,4-difluoro-benzamide	selective KCNQ2 and KCNQ3 activator^[33]^

### Quantitative RT-PCR

CPA vessels (n = 15, N = 15 normotensive controls and n = 18, N = 18 pre-eclamptic women) were cleaned of blood and placed into tubes without RNA enzymes before snap-freezing in liquid nitrogen. After thawing in ice, total RNA was extracted from arteries by homogenization in TRIzol reagent (Tiangen Biotech, Beijing, CN). RNA quality and concentration were verified respectively by gel electrophoresis and spectrophotometry using a Bio-Rad 3000 UV-vis spectrophotometer (Bio-Rad Laboratories, West Berkeley, California, USA). cDNA was synthesized with oligo (dT) primers and reverse-transcribed using a Primescript RT reagent kit (Rever Tra Ace-α-, Toyobo, Japan). Quantitative reverse-transcription PCR (RT-PCR) was performed with a Bio-Rad CFX real-time PCR system using Taq PCR Mastermix (Tiangen Biotech, Beijing, China) and SYBR Green chemistry (Toyobo CO, LTD. Japan). The conditions for amplification were as follows: 40 cycles of denaturation at 96°C for 30 sec, annealing at 57°C for 30 sec, and extension at 72°C for 30 sec. PCR was completed by final extension at 72°C for 10 min. The specificity of PCR amplification products was validated by melting curve analysis. Information on all genes of human Kv7 isoforms (Kv7.1–7.5, encoded by KCNQ1-5) is publicly available. The primers sequences used in this study are listed in Table 3. A relative quantification method was used. Ct (Cycle threshold) values were determined with CFX PCR software, and RNA abundance relative to the housekeeper gene β-actin was calculated as ΔCt. The fold-change in KCNQ gene expression between normotensive and pre-eclamptic women was then calculated as 2^-ΔΔCt^.

**Table 3:** The PCR primers used in this experiment.

Gene symbol	Primer sequences	Products size
KCNQ1	1-F:5'CGACGCATGCAGTACTTTGTGG3'1-R:5'CCAGCTGCGTCACCTTGTCTTC3'	256bp
KCNQ2	2-F:5'CGGCGGGCTTTCCTGTTCC3' 2-R:5'CTCGGCTGCAGGCGAAGGTCT3'	141bp
KCNQ3	3-F:5'GGCCCTTCTATACCCCTCTGA3' 3-R:5'GCCGACTTCACCTCTCCAACAG3'	161bp
KCNQ4	4-F:5'CCCACCCCTTTGCTCCTCTTC3’ 4-R: 5'GAGGGCATGTTGGGGCAGAG3'	205bp
KCNQ5	5-F:5'GTGACTGGGCAGGCTCTCTCCC3' 5-R:5'CTTAACGAGGCCTAAGGAGGGG3'	159bp
β-actin	F:5’CCACCATGTATCCGGGCATT3’ R:5’TGCTACGCATCTGCTGAGTC3’	244bp

### Western blotting

CPA vessels were collected from normotensive (n = 6, N = 6) or pre-eclamptic (n = 6, N = 6) women, homogenized on ice in RIPA buffer [20 mM Tris, pH 7.5, 150 mM NaCl, 1% Triton X-100, 2.5 mM sodium pyrophosphate, 1 mM EDTA, 1% Na3VO4, 0.5 μg/ml leupeptin, 1 mM phenylmethanesulfonyl fluoride (PMSF), Beyotime Biotechnology, Shanghai, CN] for 20–30 minutes. Each homogenate was centrifuged (9180xg, 4°C for 15~20 min), and the supernatants were stored at -80°C. All supernatant concentrations of protein were determined using an Enhanced BCA protein assay kit (Beyotime Biotechnology, Shanghai, CN). An equal amount of protein (80 μg/lane) was loaded onto 6% polyacrylamide-SDS gels, subjected to electrophoresis, and transferred onto polyvinylidene fluoride (PVDF) membranes. Membranes were blocked for 2 h at room temperature in blocking buffer (5% dried skim milk in 0.5% Tween-TBS (Sangon Biotech, Shanghai, CN).

Membranes were probed overnight at 4°C with primary antibodies anti-Kv7.3 (Abcam, Cambridge, UK; ab66640), anti-Kv7.4 (Abcam, Cambridge, UK; ab65797), anti-Kv7.5 (Abcam, Cambridge, UK; ab66740) at 1:500 or 1:1000 for β-Actin (Santa Cruz, CA, USA, sc-47778) in blocking buffer. Three 10-min washes in TBS/0.5% Tween 20 were followed by incubation with horseradish peroxidase-conjugated secondary antibody (goat anti-rabbit for Kv7.2-Kv7.5 and goat anti-mouse for β-Actin) at 1:1000 dilution for 2 h. After three 10-min washes in TBS/0.5% Tween 20, membranes were developed by Chemiluminescence reactions, then detected on a Tanon-2000 Gel imaging system (Tanon Science & Technology Co., Ltd, Shanghai, CN). Each band was quantified using a TU-1900 ultraviolet spectrophotometer (Purkinje General Instrument co., LTD, Beijing, CN). Relative expression levels were normalized relative to the levels of β -actin.

### Data analysis

The effects of XE991 on arterial basal tone and contraction induced by U46619 were expressed as a percentage of the contraction to 80 mM Tyrode’s solution. Relaxation data were expressed as a percentage of the maximum contraction to U46619 (10^−7^ M). Contraction and relaxation response curves were subjected to transformation to allow comparison by two-way ANOVA. Significance of the differences between groups was assessed using two-way ANOVA followed by the least significant difference (LSD) post hoc test when appropriate. All results were reported as the mean ± SD. All analyses and plots were performed with either Graph Pad Prism 5.0 or SPSS 11.0 software.

## Results

### Functional experiments on CPAs from normotensive women

**Fig 1:**
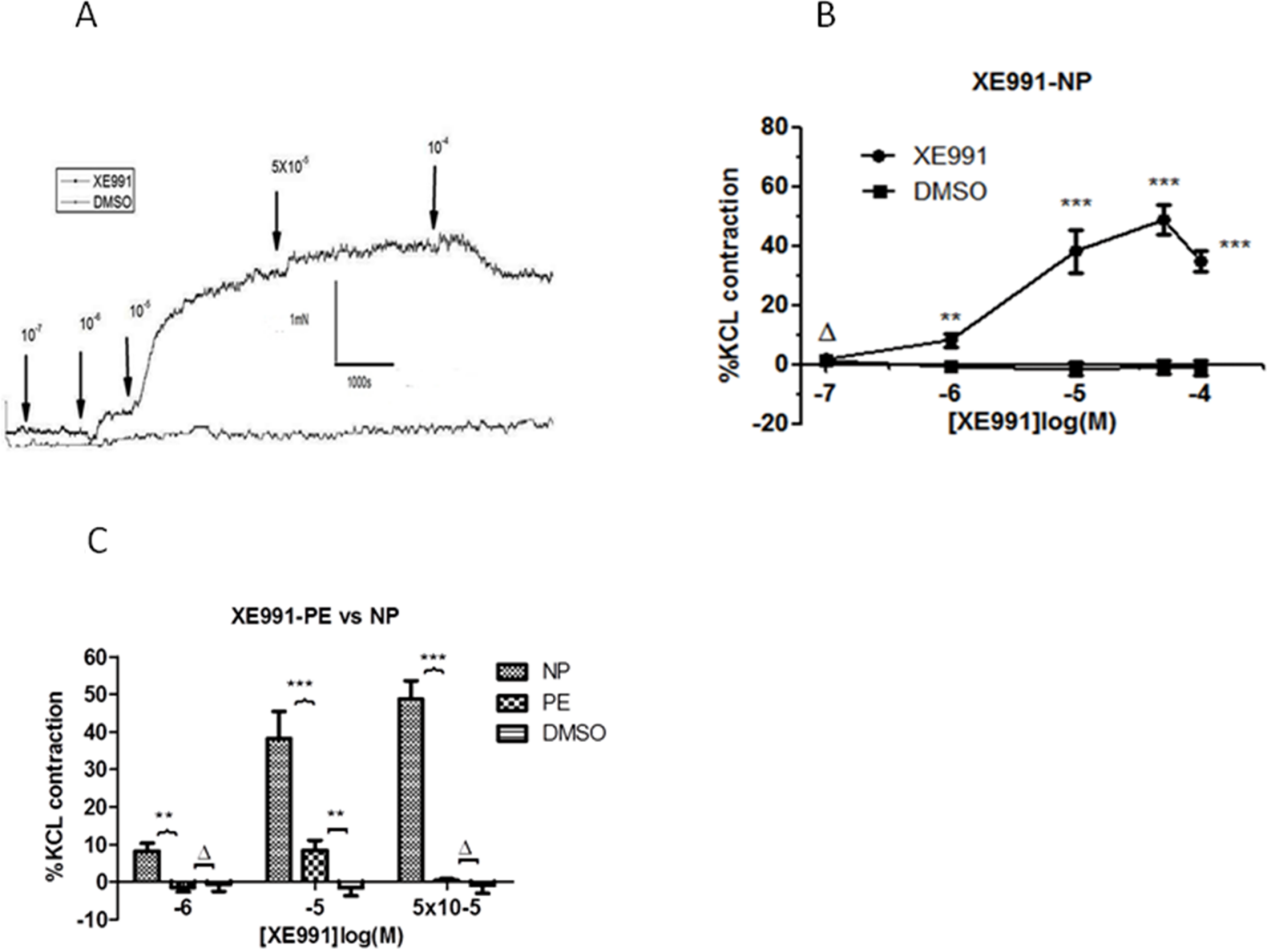
Effect of Kv7 channel blocker XE991 on baseline tone of chorionic plate arteries. (A) The representative tracings of changes in tension of the chorionic plate arterial ring from normotensive women in response to the cumulative addition of XE991 (Kv7 channel blocker) from 10^-7^mol/L to 10^-4^mol/L compared with dimethyl sulfoxide (DMSO) treated controls. (B)Effect of XE991 (Kv7 channel blocker, 10^−7^–10^−4^ mol/L) on baseline tone of chorionic plate arteries from normotensive women (n = 10 vessels, N = 10 placentas), compared with dimethyl sulfoxide (DMSO) treated controls. ΔP>0.05, **P<0.01, ***P<0.001 vs. Control (two-way ANOVA). (C) Comparison of effects of XE991 (10^−6^, 10^−5^,5x10^-5^M/L) on baseline tone of chorionic plate arteries from preeclampsia women (n = 10 vessels, N = 10 placentas) compared with normotensive women or DMSO controls (n = 10 vessels, N = 10 placentas) (ΔP>0.05,**P<0.01, ***P<0.001, two-way ANOVA).

**Fig 2:**
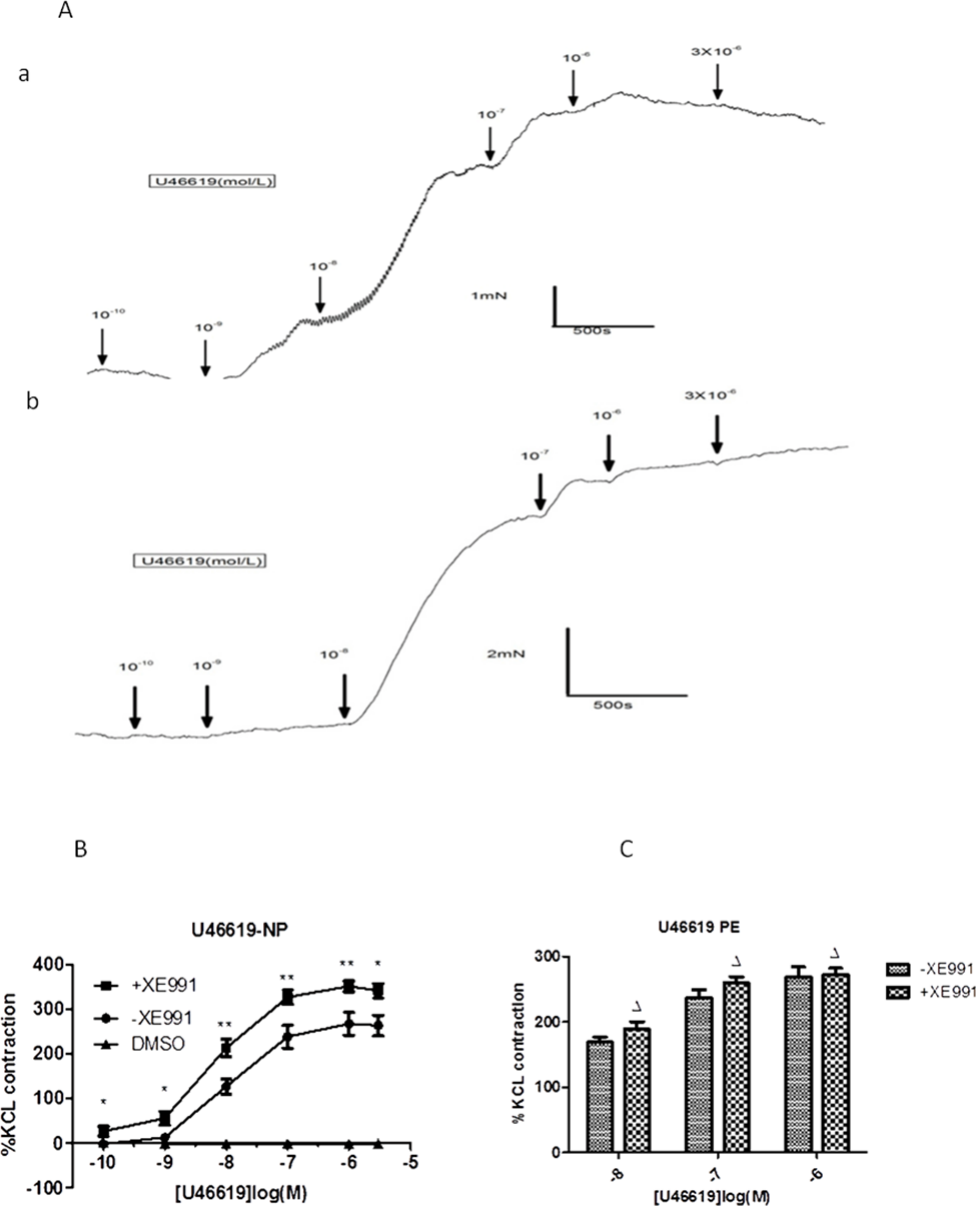
Contraction effects of U46619 in the presence and absence of XE991 (10^−5^ M/L). (A)The representative tracings of changes in tension of the chorionic plate arterial ring from normotensive women in response to the cumulative addition of U46619 (thromboxane receptor agonist) from 10^-10^mol/L to 3X10^-6^mol/L. The panel a shows the response to U46619 without incubation of XE991(10^−5^ M/L) The panel b shows the response to U46619 in the presence of XE991(10^−5^ M/L) (B) Concentration response curves(mean±SD; n = 10 vessels, N = 10 placentas) for U46619(10^−10^-3X10^-6^ mol/L) in the presence and absence of XE991(10^−5^ M/L) on the chorionic plate arteries from normotensive women.(*P<0.05, **P<0.01, +XE991 vs.–XE991, two-way ANOVA). (C) Comparison of contraction effects of U46619 (10^−8^–10^−6^ mol/L) on chorionic plate arteries from preeclampsia women in the presence and absence of XE991 (10^−5^ M/L). (ΔP>0.05, +XE991 vs.–XE991, two-way ANOVA).

Kv7 channels participated in regulating vascular basal and agonist-induced tone, as found in the following experiments: 1) the Kv7 channel broad spectrum blocker XE991(10^−7^–10^−4^ mol/L)increased arterial basal tone. Fig 1A shows that XE991 produced a concentration-dependent contraction, which was contrasted by application of DMSO (dimethyl sulfoxide, the solvent of XE991). Fig 1B shows the concentration-response curve (n = 10, N = 10, where n is the number of vessels and N is the number of cases), and a two-way ANOVA comparing XE991 and dimethylsulfoxide (DMSO) at each treatment concentration showed significance. The most significant change was achieved at 5×10^−5^ mol/L, and the mean contraction at this concentration had an amplitude 48.67±15.60% of that induced by 80 mM K^+^. Upon addition of 10^−4^ mol/L, vessels displayed relaxation, with tension amplitude returning approximately 34.91±11.08% towards the baseline. 2) A significantly increased U46619(10^−10^-3X10^-6^ mol/L)-induced contraction of CPAs was observed in the presence of 10^−5^ mol/L XE991 (n = 10, N = 8), with each concentration showing significance,*P<0.05, **P<0.01, vs. XE991-absent control (Fig 2C). Fig 2A and 2B show the representative tracings of changes in tension of the chorionic plate arterial ring from normotensive women in response to the cumulative addition of U46619 (thromboxane receptor agonist).

**Fig 3:**
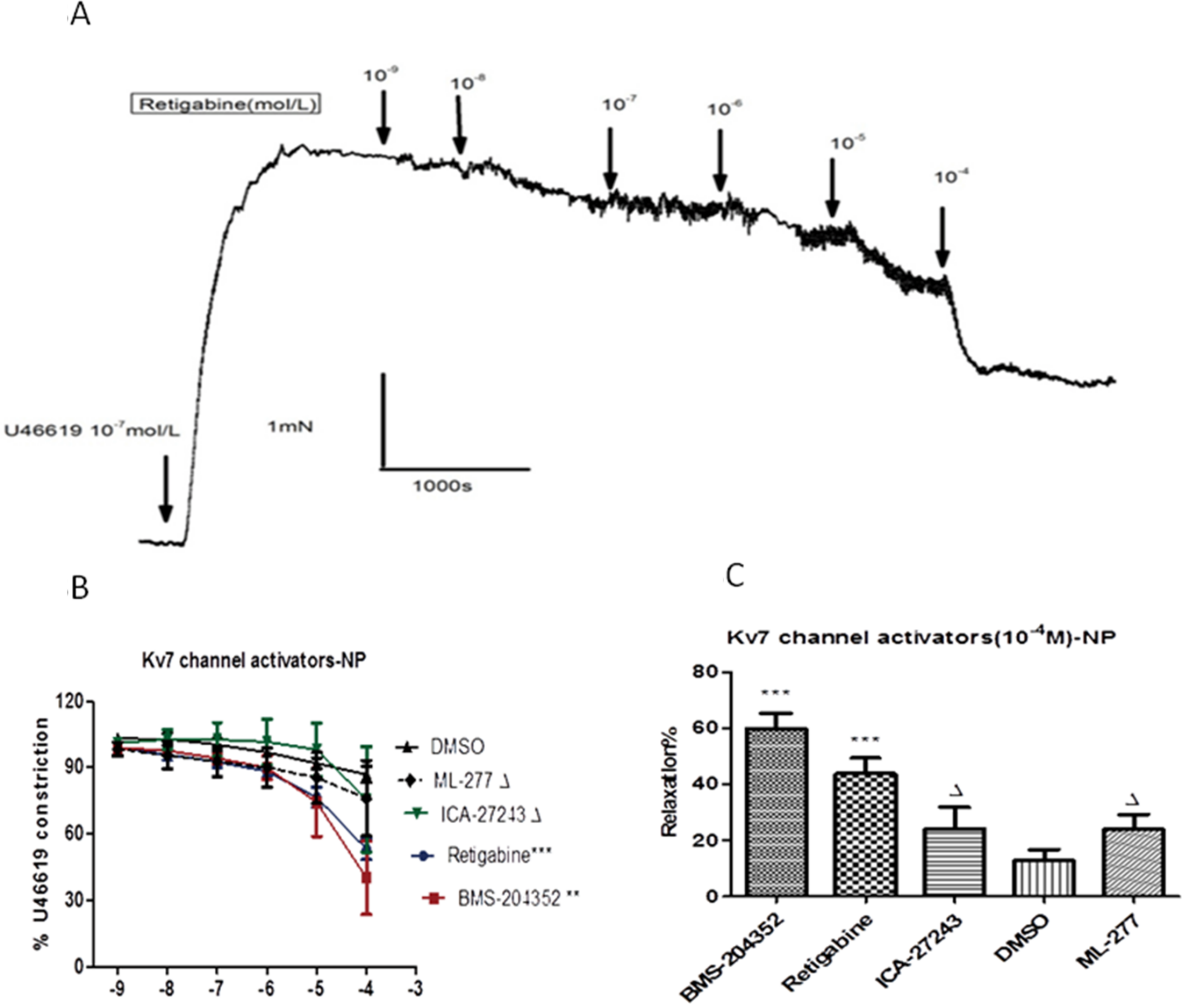
The relaxation effect of Kv7 activators in chorionic plate arteries from normotensive women. (A) The representative tracings of changes in tension of the chorionic plate arterial ring from normotensive women in response to the cumulative addition of retigabine (Kv7.2–7.5 activator) from 10^−9^ mol/L to 10^-4^mol/L. The ring was contracted with U46619(10^-7^mol/L) (B)Concentration effect curves for each Kv7 activator(10^−9^~10^-4^mol/L) on chorionic plate arteries from normotensive women (n = 10 vessels, N = 10 placentas), compared with DMSO treated controls. ΔP>0.05, **P<0.01, ***P<0.001, vs. control (two-way ANOVA followed by Least Significant Difference (LSD) post hoc test). (C) Comparison of the effect of the four Kv7 activators (10^-4^mol/L) in chorionic plate artery from normotensive women, compared with DMSO treated controls. (ΔP>0.05, ***P<0.001, vs. control).

The functional impact of Kv7 channels in agonist-induced CPA contraction was also demonstrated with a range of Kv7 activators (retigabine, BMS-204352, ML277 and ICA-27243, 10^−9^~10^−4^ mol/L). Fig 3A shows the representative tracings of changes in tension of the chorionic plate arterial ring from normotensive women in response to the cumulative addition of retigabine (Kv7.2–7.5 activator). Fig 3B shows that BMS-204352 (Kv7.2–7.5 activator) (n = 9, N = 9) and retigabine (n = 10, N = 8) produced a concentration-dependent relaxation in the U46619 (10^−7^ mol/L) pre-contracted CPAs, with two-way ANOVA tests comparing the Kv7 activators and DMSO, **P<0.01, ***P<0.001. In contrast, ICA-27243 (selective opener of Kv7.2 and Kv7.3) (n = 10, N = 7) and ML277 (selective Kv7.1 activator) (n = 10, N = 7) had no significant effects on tension in the pre-contracted CPAs (ΔP>0.05, ΔP>0.05, two-way ANOVA). Fig 3C shows that the contraction of CPAs was relaxed by >50% with application of BMS-204352 (10^−4^ mol/L), which induced the greatest relaxation to 59.87±16.49% (***P<0.001 vs. DMSO control). Retigabine (10^−4^ M) relaxed pre-contracted vessels by 43.92±17.21% (***P<0.001 vs. DMSO control), ICA-27243(10^-4^M) and ML277 (10^-4^M) relatively had no effect in tension of the pre-contracted CPAs (ΔP>0.05 vs. DMSO control).

### Functional experiments using CPAs from pre-eclamptic women

Fig 1B shows obvious contraction effects of the broad spectrum channel blocker XE991 on vascular basal tone in normotensive CPAs, while the effects of XE991 (10^−6^, 10^−5^, 5x10^-5^ M/L) on the contraction of CPAs from pre-eclamptic women were considerably attenuated (n = 10, N = 7, normotensive vs. pre-eclampsia by two-way ANOVA, **P<0.01, ***P<0.001, Fig 1C). Similarly, the percentage point difference of U46619-induced contraction in the presence and absence of XE991 (10^−5^ M) is shown in Fig 2D (n = 10, N = 6, normotensive vs. preeclampsia by two-way ANOVA, ΔP>0.05). The potent contraction effect of XE991 (10^−5^ M/L) on U46619 (10^−8^, 10^−7^, and 10^−6^ M/L)-induced contraction was almost abrogated in CPAs from pre-eclamptic women.

**Fig 4:**
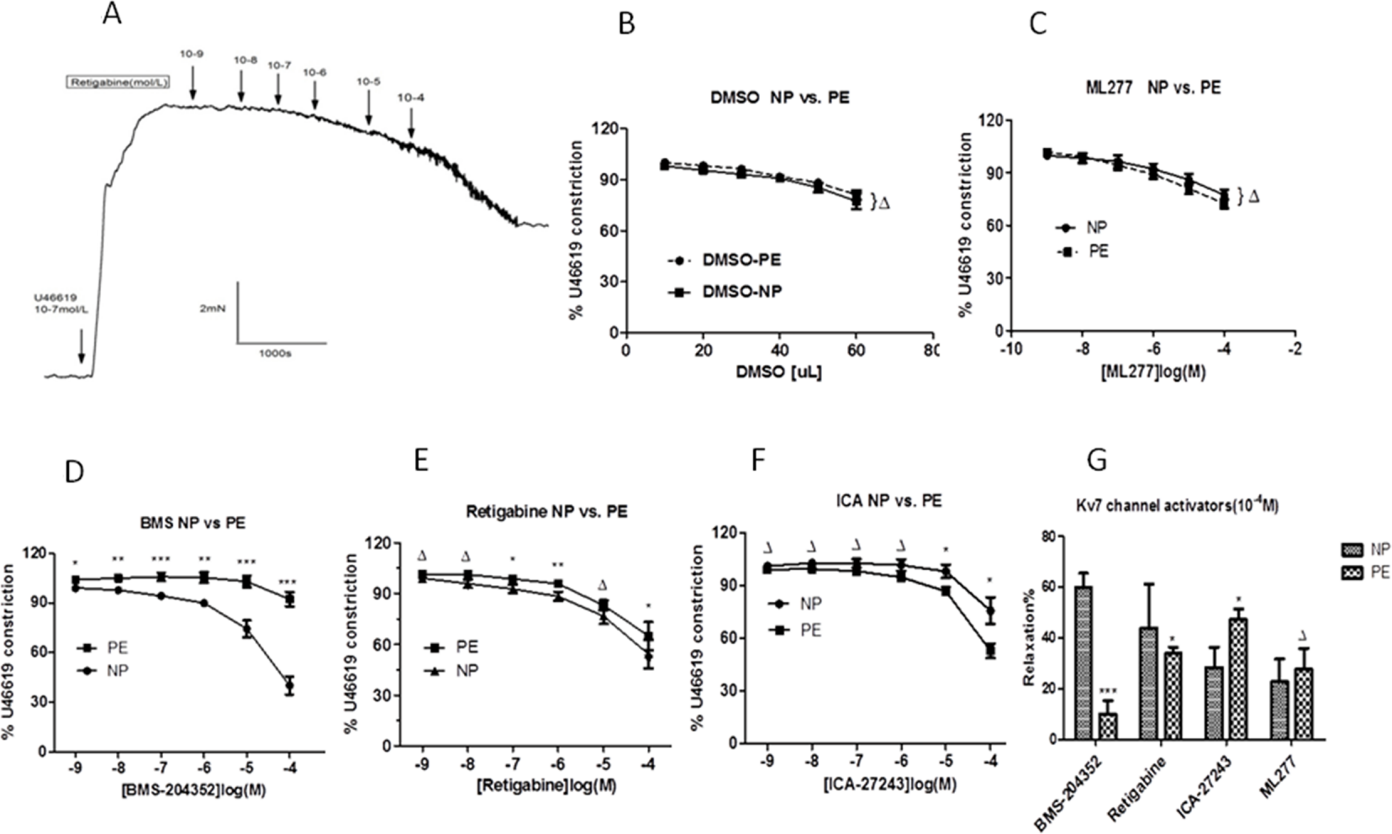
The relaxation effect of Kv7 activators in chorionic plate arteries from normotensive controls and pre-eclampsia women. (A) The representative tracings of changes in tension of the chorionic plate arterial ring from preeclampsia women in response to the cumulative addition of retigabine (Kv7.2–7.5 activator) from 10^−9^ mol/L to 10^-4^mol/L. The ring was contracted with U46619 (10^-7^mol/L). (B) Comparison of the concentration effect curves for DMSO(10~60uL) in chorionic plate arteries from preeclampsia women(n = 10 vessels, N = 10 placentas) compared with normotensive controls (n = 10 vessels, N = 10 placentas).(C-F)Comparison of the concentration effect curves for 4 Kv7 channel activators(10^−9^~10^-4^mol/L) on chorionic plate arteries from preeclampsia women(n = 10 or 14 vessels, N = 10 or 14 placentas) compared with normotensive controls(n = 10 vessels, N = 10 placentas). (G) Comparison of the effects of 4 Kv7 channel activators (10^-4^mol/L) in preeclampsia women compared with normotensive controls. ΔP>0.05, *P<0.05, **P<0.01,***P<0.001,two-way ANOVA followed by Least Significant Difference (LSD) post hoc test.

In addition to the reduced effectiveness of Kv7 channel blockers, the ability of the Kv7 channel activators to relax agonist-induced CPA vessels from pre-eclamptic women was significantly altered. Fig 4A shows the representative tracings of changes in tension of the chorionic plate arterial ring from preeclampsia women in response to the cumulative addition of retigabine (Kv7.2–7.5 activator). Fig 4D and 4E shows that the effect of BMS-204352 (n = 9, N = 7) and retigabine (n = 14, N = 7) on the relaxation of pre-contracted CPA vessels from pre-eclamptic women was notably down-regulated (ΔP>0.05,*P<0.05,**P<0.01,***P<0.001 vs. normotensive, two-way ANOVA), especially the relaxation of BMS-204352 was obviously altered. Interestingly, the relaxation ability of ICA-27243 on pre-contracted CPA vessels from pre-eclamptic women (n = 10, N = 7) was enhanced (Fig 4F,Δ>0.05,*P<0.05, two-way ANOVA). Fig 4G shows the effect of Kv7 channel activators (10^−4^ M) on the pre-contracted CPA vessels of pre-eclampsia women. Application of BMS-204352 (10^-4^M) and retigabine (10^-4^M) only relaxed 12.43±7.85% and 35.10±8.25% of the vessel contraction in the pre-eclampsia CPAs, respectively, compared with relaxations of 59.87±16.49% (***P<0.01) and 43.92±17.21% (*P<0.01) observed in the normotensive CPAs, respectively. ICA-27243 (10^-4^M) relaxed contraction of pre-contracted CPAs from pre-eclamptic women to 47.20±13.04%, compared with relaxation of 22.32±15.48% observed in the normotensive CPAs (*P<0.05).

### Expression profiles of KCNQ subunits in CPAs from normotensive controls and pre-eclamptic women

**Fig 5:**
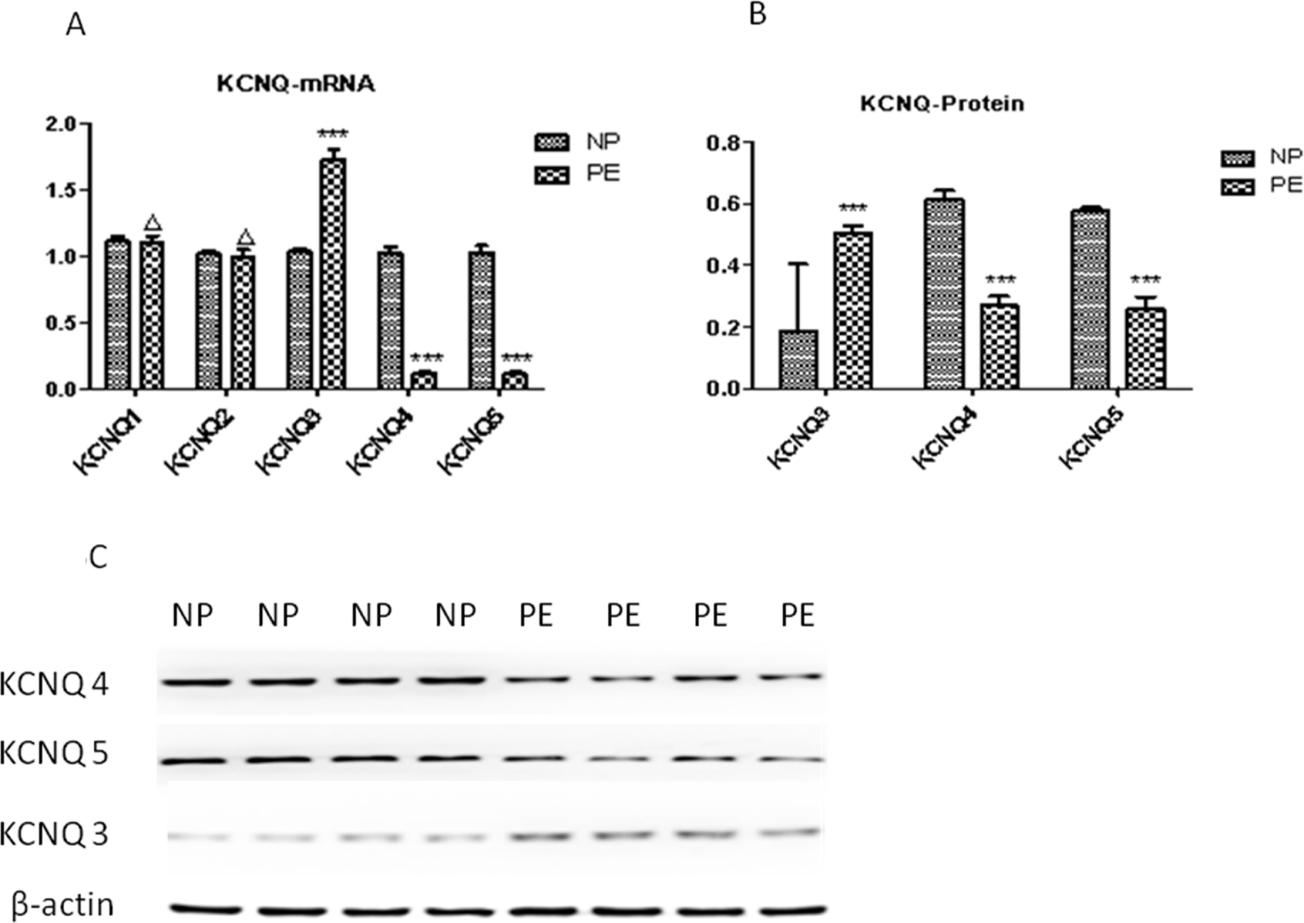
Expression profiles of KCNQ subunits in preeclamptic women compared with normotensive controls. (A) Quantitative polymerase chain reaction analysis of relative abundance of KCNQ genes 1 through 5. Data are normalized to the first normotensive control. (B) Densities of Kv7.3, 7.4, 7.5 bands normalized to their respective β-actin bands. (C).Representative Western blot for Kv7.3, 7.4, and 7.5 expression in total protein lysates run on the same gel. The respective concentrations of protein loaded were identical, as illustrated by theβ-actin bands. ΔP>0.05, ***P<0.001, according to independent-samples t test.

RT-qPCR was undertaken to explore whether impairment of Kv7 function in the pre-eclampsia CPAs was due to reduced expression of KCNQ genes. Expression of KCNQ1 and KCNQ2 remained largely unchanged (N = 14 to 18, P>0.05, P>0.05), whereas expression of KCNQ4 and KCNQ5 markedly decreased in CPAs from pre-eclamptic women compared with normotensive control CPAs (Fig 5A, Table 4). In contrast, expression of KCNQ3 unexpectedly increased in CPAs from pre-eclamptic women compared with normotensive control CPAs (Fig 5A, Table 4). Because the mRNA levels of KCNQ genes 3–5 were markedly altered in CPAs from pre-eclampsia women compared with CPAs from normotensive women, we investigated whether this difference was maintained at the protein level. Densitometric analysis of protein bands in the CPAs from normotensive women versus those from pre-eclamptic women were normalized to their respective β-actin bands. The density of bands for KCNQ3 in pre-eclamptic women was significantly increased to 0.4997±0.0422, while the density of bands for KCNQ3 in normotensive control women was 0.1854±0.0368 (n = 6, N = 6, p<0.001). In addition, the density of bands for KCNQ4 and KCNQ5 in pre-eclamptic women was significantly decreased to 0.2572±0.0705 (n = 6, N = 6, p<0.001) and 0.2626±0.0567 (n = 6, N = 6, p<0.001), respectively, while the density of bands for KCNQ4 and KCNQ5 in the normotensive women was 0.5768±0.0181 and 0.5987±0.0492, respectively (Fig 5B and 5C).

**Table 4:** Comparison of the relative mRNA expression of KCNQ genes 1–5 between the normotensive pregnancy and pre-eclampsia groups.

Kv7 genes	PE group(n = 18)	NP group (n = 15)	P
KCNQ1	1.1039±0.0508	1.1148±0.0351	P>0.05
KCNQ2	0.9968±0.0541	1.0172±0.0247	P>0.05
KCNQ3	1.7341±0.0696	1.0337±0.0224	P<0.001
KCNQ4	0.1113±0.0115	1.0223±0.0473	P<0.001
KCNQ5	0.1110±0.0134	1.0280±0.0510	P<0.001

## Discussion

Our study find that functional Kv7 channels in human CPAs since firstly XE991 significantly increased basal and agonist-induced tone and secondly a range of specific Kv7 activators with key differences in chemical scaffolds significantly relaxed precontracted CPAs, which is consistent with observations in systemic vascular tissues from animals and humans [22–27]. Similar to reference 27 we show that Kv7 activators are effective relaxants of precontracted CPAs. Ng et al found that the Kv7 channel blockers XE991 increased isometric tension and reversely the two Kv7 channel activators, retigabine and acrylamide S-1, relaxed preconstricted human arteries(visceral adipose tissue and mesenteric arteries), actions reversed by XE991[26].In addition, the relaxation induced by different Kv7 channel activators on the CPA vessels suggests a potential role for these channels as targets in pregnant women with increased fetoplacental vascular resistance, such as in preeclampsia. However, the most striking finding is that the functional impact of Kv7 channels on vascular tension was dramatically reduced in CPA vessels from preeclamptic women. Namely, the effect of Kv7 channel blocker XE991 on basal tone and agonist (U46619)-induced contraction was markedly attenuated in preeclamptic women’s CPA vessels compared to those of normotensive women. In addition, the relaxant effects of the Kv7.2–7.5 activators BMS-204352 and Retigabine were diminished in CPA vessels from preeclamptic women, and the effect of BMS-204352 was almost entirely abolished. ICA-27243, a selective KCNQ2 and KCNQ3 activator [33], had no effect on pre-contracted CPA vessels of normotensive women, but its ability to relax pre-contracted CPA vessels was interestingly enhanced in pre-eclamptic women. Furthermore, ML277, a selective KV7.1 activator [32], had relatively no effect on tension in the pre-contracted CPAs in our study, even though KCNQ1 mRNA was present in the CPA vessels. This decreased effect of Kv7 channels on vascular tension was not unexpected since Jepps et al first showed that Kv7 activity was reduced in two models of hypertension [34]. This was extended to include renal artery [34] and coronary artery [35]. In each of these cases Kv7.4 protein was reduced without a decrease in KCNQ4 transcript. Kv7 channel activity was also compromised in coronary arteries of diabetic rats [36],penile arteries from rats with early metabolic disease [37] and pulmonary artery hypertension [38]. In another aspect, this is the first exploration of altered KCNQ subunit expression in the CPA vessels of women with pre-eclampsia to our knowledge. Our data demonstrate that mRNA levels for KCNQ3 were specifically up-regulated, whereas those for KCNQ4 and KCNQ5 were down-regulated in CPA vessels from pre-eclamptic women compared with those in normotensive controls. Similar observations were found in subsequent probing of protein levels of KCNQ genes 3–5 in the CPA vessels from preeclamptic women. Thus, our present study shows that the transformation of Kv7 channel function in CPAs of preeclamptic women may be associated with considerably altered expression profiles of Kv7 subunits. Moreover, these results imply that the impaired function of Kv7 channels and altered expression of KCNQ isoforms in CPA vessels from preeclamptic women are concomitant with the hypertensive state of preeclampsia, which contributes to the increased vascular resistance of CPAs followed by reduced fetoplacental blood flow.

Research over the past several decades has demonstrated an important role for Kv7 channels in regulation of the excitability of vascular smooth muscle [22–26] as well as nonvascular tissues, including a role in neurons and cardiomyocytes [39–40]. In all previous studies mentioning the expression of Kv7 channel subunits in vascular tissues, it has consistently been found that KCNQ1, KCNQ4, and KCNQ5 were the most abundantly expressed isoforms, with a relatively minor contribution from KCNQ genes 2–3 and occasionally undetectable KCNQ2 levels [22–27]. The accumulated evidence has suggested that KCNQ4 and KCNQ5 channel isoforms play a predominant role in the regulation of the excitability of vasculature. Furthermore, the previously demonstrated relaxation effects of the novel anxiolytic BMS-204352 (Maxipost, a Kv7.2-Kv7.5 opener) might be more predominantly mediated by Kv7.4 and Kv7.5 channels than retigabine (an antiepileptic drug, a Kv7.2–5 opener) in the mesenteric artery [41]. Therefore, in this study, significantly decreased relaxation effects of BMS-204352 and Retigabine and markedly attenuated effects of the Kv7 channel blocker XE991 on basal tone and agonist (U46619)-induced contraction might be predominantly mediated by the considerably lower expression of KCNQ4 and KCNQ5 found in CPA vessels from preeclamptic women.

An interesting finding of the present study was the increased effect of ICA-27243 in CPA from preeclamptic woman. As arteries from these women exhibited increased expression of KCNQ3 we speculate the up-regulation of KCNQ3 in preeclampsia may lead to activation of those channels, thus enhancing their function following significant relaxation of ICA-204352 on CPA vessels in preeclampsia. For all this, because of the reduced effect of XE991 in PE arteries despite increased expression of KCNQ3, the up-regulation of KCNQ3 isoforms could be viewed as a compensatory mechanism for the reduced placental perfusion of preeclamptic women caused by considerable down-regulation of KCNQ4 and KCNQ5. Thus,the potential use of drugs such as ICA-27243 might be effective vasodilators in preeclamptic subjects despite being less effective in normal subjects.

Our present study is not only the first to provide an extensive comparison of the Kv7 channel function effects and the correlating altered expression of KCNQ subunits in CPAs taken from pre-eclamptic women compared with normotensive controls, but it also reveals that altered Kv7 channels may contribute to the vascular pathophysiology of preeclampsia. Despite this, it remains unknown whether altered Kv7 channels are the initial factor necessary for the occurrence of pre-eclampsia, and further studies are needed. Since the functional impact of Kv7 channels on the CPA vessels was identified through measurement of isometric tension in vitro, further work is required to assess the effects of Kv7 regulators by patch clamp electrophysiological experiments at the cellular level, and in vivo with animal models of preeclampsia. Our results indicate that Kv7.3, Kv7.4 and Kv7.5 subunits deserve attention in the regulation of CPA vascular function in preeclamptic women, especially up-regulation of the Kv7.3 subunit, which should be further confirmed by electrophysiological studies.

## Supporting information

S1 File The clinical data of all participating subject (Table 1). (XLSX)

S2 File Effect of Kv7 channel blocker XE991 on baseline tone of chorionic plate arteries. The percentage changes of CPAs basic tension from Normotensive vs. DMSO (Fig 1B) and Normotensive vs. preeclampsia (Fig 1C).
(XLSX)

S3 File Contraction effects of U46619 in the presence and absence of XE991 (10^−5^ M/L). The contraction percentages of U46619 in the presence and absence of XE991 (10–5 M/L) on CPAs from normotensive (Fig 2C) and preeclampsia (Fig 2D).
(XLSX)

S4 File The relaxation effect of Kv7 activators in chorionic plate arteries from normotensive women. The Percentage changes of each Kv7 activator(10–9~10–4mol/L) to U46619(10–7mol/L) induced-contraction in chorionic plate artery from normotensive women(Fig 3B and 3C and Fig 4B–4G).
(XLSX)

S5 File The relaxation effect of Kv7 activators in chorionic plate arteries from normotensive controls and pre-eclampsia women. The Percentage changes of each Kv7 activator(10–9~10–4mol/L) to U46619(10–7mol/L) induced-contraction in chorionic plate arteries from preeclampsia women(Fig 4B–4G).
(XLSX)

S6 File Expression profiles of KCNQ subunits in pre-eclampsia women compared with normotensive controls. The relative expression quantity of mRNA (Fig 5A) and protein (Fig 5B) of KCNQ genes (normotensive = NP, preeclampsia = PE).
(XLSX)
